# 50 Years of structural immunology

**DOI:** 10.1016/j.jbc.2021.100745

**Published:** 2021-05-03

**Authors:** Ian A. Wilson, Robyn L. Stanfield

**Affiliations:** 1 Department of Integrative Structural and Computational Biology, The Scripps Research Institute, La Jolla, California, USA; 2 The Skaggs Institute for Chemical Biology, The Scripps Research Institute, La Jolla, California, USA

**Keywords:** immune recognition, humoral immunity, cellular immunity, antibodies, MHC, T cells, TLR, VLR, microbial pathogens, viral antigens, CDR, complementarity-determining region, CoV, coronavirus, EPO, erythropoietin, EPOR, erythropoietin receptor, GP, glycoprotein, HA, hemagglutinin, hGH, human growth hormone, Ig, immunoglobulin, MHC, major histocompatibility complex, MR, molecular replacement, NA, neuraminidase, NKT cell, natural killer T cell, NTD, N-terminal domain, PDB, Protein Data Bank, RBD, receptor-binding domain, RSV, respiratory syncytial virus, TCR, T cell receptor, TLR, Toll-like receptor, VLR, variable lymphocyte receptor

## Abstract

Fifty years ago, the first landmark structures of antibodies heralded the dawn of structural immunology. Momentum then started to build toward understanding how antibodies could recognize the vast universe of potential antigens and how antibody-combining sites could be tailored to engage antigens with high specificity and affinity through recombination of germline genes (V, D, J) and somatic mutation. Equivalent groundbreaking structures in the cellular immune system appeared some 15 to 20 years later and illustrated how processed protein antigens in the form of peptides are presented by MHC molecules to T cell receptors. Structures of antigen receptors in the innate immune system then explained their inherent specificity for particular microbial antigens including lipids, carbohydrates, nucleic acids, small molecules, and specific proteins. These two sides of the immune system act immediately (innate) to particular microbial antigens or evolve (adaptive) to attain high specificity and affinity to a much wider range of antigens. We also include examples of other key receptors in the immune system (cytokine receptors) that regulate immunity and inflammation. Furthermore, these antigen receptors use a limited set of protein folds to accomplish their various immunological roles. The other main players are the antigens themselves. We focus on surface glycoproteins in enveloped viruses including SARS-CoV-2 that enable entry and egress into host cells and are targets for the antibody response. This review covers what we have learned over the past half century about the structural basis of the immune response to microbial pathogens and how that information can be utilized to design vaccines and therapeutics.

As we celebrate 50 years of the Protein Data Bank (PDB), it is fitting to start this review with a reflection on the birth of structural immunology that began with landmark papers on antibody structures published 50 years ago in 1971 (1, 2). It was a different time back then when pure proteins were much harder to obtain and structures were equally hard to determine. As structural methods, technologies, and computing improved and recombinant protein expression became possible, the opportunities to tackle previously intractable problems in structural immunology, as well as in structural biology in general, exploded to where we are today with a comprehensive understanding of how microbial pathogens are recognized and countered by the immune system. The PDB played a pivotal role in this whole process by collating and curating the structures that could facilitate structure determination of a macromolecule of choice by molecular replacement. The PDB also enabled mining of the rich arsenal of structural data that allowed general principles for immune recognition to be identified and then harnessed for structure-based design of vaccines and therapeutics. In this review, we provide examples and share our thoughts on how structural biology has shaped our understanding of immune receptors and how they function.

## Antibody structure

**Figure 1:**
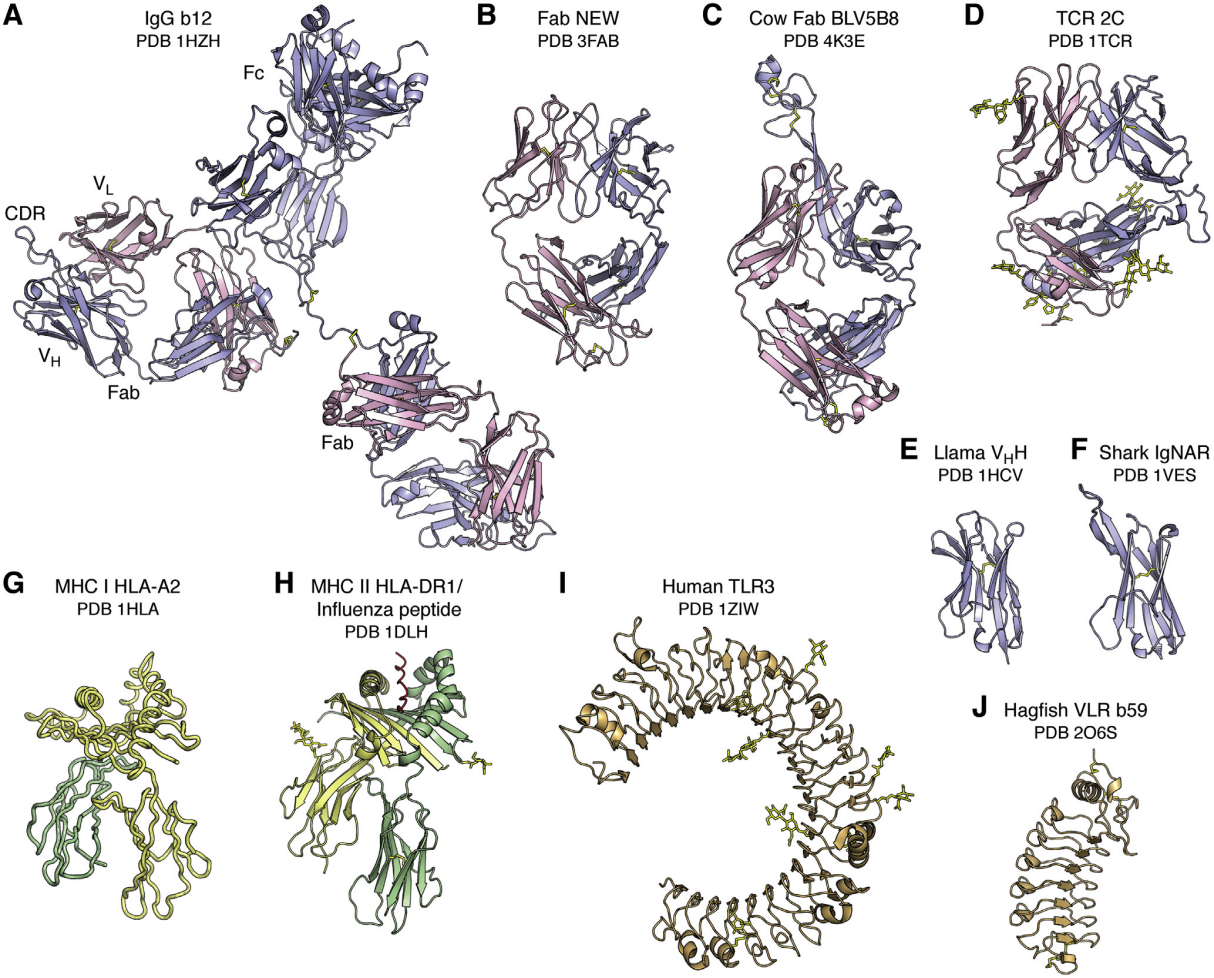
**The diversity of antigen receptors in the immune system.** The immunoglobulin fold is utilized as the recognition motif in antibodies (*A*, *B*, *C*, *E* and *F*) in the humoral adaptive immune system and T cell receptors (*D*) in the cellular adaptive immune system. The intact IgG b12 (*A*) is labeled to illustrate the relative positions of the two Fab and one Fc regions, the V_L_ and V_H_ immunoglobulin domains within one Fab, and the complementarity-determining region containing region of the Fab. The camelid family (*E*), which includes llamas, and the shark family (*F*), also have smaller antibodies that contain only a single V_H_ domain (termed V_H_H or nanobody) instead of the two V_H_ and V_L_ domains in conventional antibodies. The major histocompatibility complex (MHC) fold (*G* and *H*) is used to present peptide antigens to T cell receptors for classical MHC I and II, and lipids, glycolipids, specialized peptides, and other antigens in nonclassical MHC-like molecules. Note that, in (*G*), the unrefined HLA-A2 structure was deposited with only Cα atoms, so a cartoon trace is shown. Toll-like receptors (*I*) in the innate immune system adopt a Leu-rich repeat fold and recognize specific antigens, including proteins, nucleic acids, lipopolysaccharides, unmethylated CpG, and small molecules. Variable lymphocyte receptors (*J*) (VLRs) also are composed of Leu-rich repeats and function as the adaptive immune responses in the jawless vertebrates, lampreys, and hagfish. In this and following figures, the immunoglobulin light and heavy chains are colored *pink* and *light blue*. The MHC Class I heavy chain is colored *yellow* and β_2_ microglobulin chain in *green*, whereas the MHC Class II α and β chains are colored *yellow* and *green*. The TLRs and VLRs are colored in *beige*. For all figures, carbohydrate and disulfide bonds are colored *yellow*. The name of the receptor, ligand if any, and Protein Data Bank (PDB) ID are shown below each figure.

The immunoglobulin (Ig) molecule is the major antibody recognition receptor of the humoral immune system. The chemical nature of antibodies, including the different fragments (Fab, Fc) (Fig. 1), the two-chain structure (heavy and light chains), and the antibody Y shape, was first revealed by Gerald Edelman and Rodney Porter in late 1950s and in subsequent papers, for which they received the Nobel prize in Physiology or Medicine in 1972 (see https://www.nobelprize.org/prizes/medicine/1972/porter/lecture/ and https://www.nobelprize.org/prizes/medicine/1972/edelman/lecture/). Porter showed that one of the three antibody fragments that were isolated after papain digestion of rabbit antibodies was able to crystallize, and it was later appropriately named Fc for Fragment crystallizable (3).

**Figure 2:**
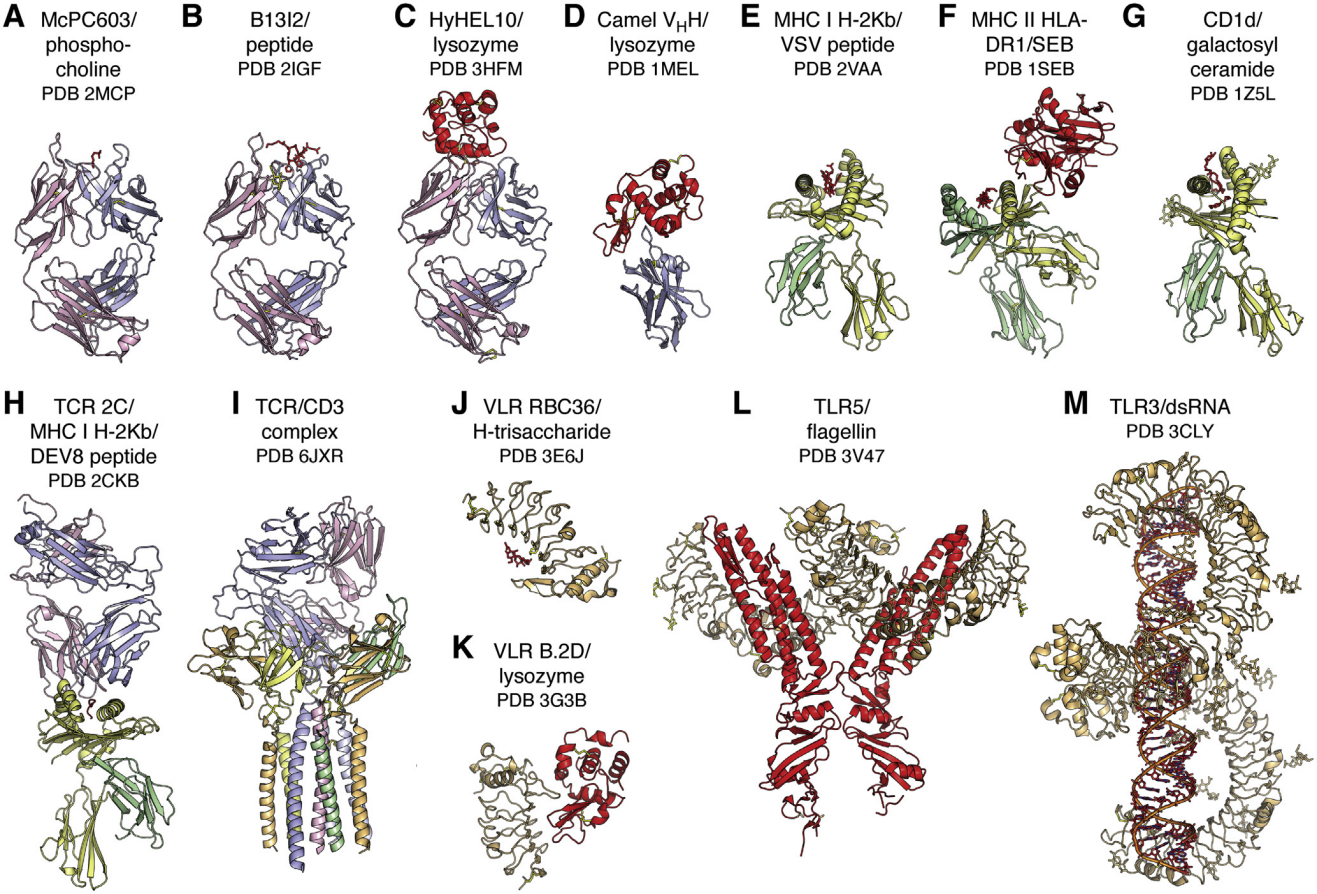
**Diversity of antigens recognized by antigen receptors in the immune system.** The diverse antigen receptors in the immune system can detect, interact with, and respond to the universe of potential antigens, shown here colored in *red* and identified under the name of its receptor. Antibodies (*A*–*D*) can recognize virtually any antigen whether large or small, and which can have diverse chemical compositions from small molecules (*A*) to carbohydrates to lipids to peptides (*B*) to proteins (*C* and *D*) and combinations thereof. Classical major histocompatibility complex (MHC) molecules (*E* and *F*) bind processed peptide antigens in their binding groove for presentation to T cell receptors (TCRs). CD1 (*G*) is related to class I MHC but presents lipid-containing antigens to certain types of T cells and NK cells. TCR signaling is initiated by binding to MHC-peptide complexes (*H*). The T cell–bound TCRs associate with the multisubunit, membrane-spanning CD3 (*I*). Variable lymphocyte receptors (*J* and *K*) and Toll-like receptors (*L* and *M*) can sense specific types of antigens present on microbial pathogens in the innate immune system and regulate inflammatory responses and transcriptional events through antigen-induced signal transduction pathways. Molecules are colored as in (*A*). In (*I*), the CD3 complex is colored with ζ chains in *blue*-*white*, δ chain in *pale green*, ε chains in *light orange*, and γ chain in *pale yellow*.

The question then was how these heavy and light chains and their substructures were arranged in molecular detail and how they bound antigen. The answers to these questions came in 1971 and 1972 with landmark structures at 6 Å resolution of two human myeloma proteins that could be isolated from serum where they were produced in excess: an intact human IgG1 called Dob (1) and the Fab' of human IgG1 New (2) (Fig. 1) from David Davies and Roberto Poljak and colleagues, respectively. This IgG antibody with a deletion in the hinge region was T-shaped and 2-fold symmetric (1). A balsa wood model to represent the structure was built (see ref. (1) for a photo of the model), and the structure was computationally modeled later in 1977 by fitting of Fab and Fc coordinates into the 6 Å electron density map (4). Very few of these early structures were deposited immediately to the PDB; however, many later became available as refined and sometimes higher-resolution structures. Structures of Fab' New with antigen (vitamin K_1_OH) at 3.5 Å followed shortly thereafter (5) as well as a higher-resolution structure to 2 Å (PDB ID: 7FAB) (6). Structures soon followed for “Bence Jones” immunoglobulin light chain dimers that are excreted into the urine of patients with multiple myelomas and were first discovered and studied by Dr Henry Bence Jones in the late 1840s (7). Individual Bence Jones proteins, such as Mcg and REI, are code named for the patient from whom they were derived. These early structures included the Bence Jones light chain dimer Mcg at 3.5 Å resolution (8) and a dimer of the REI Vκ domains at 2.0 Å (PDB ID: 1REI) (9). Another IgG structure (Kol) was determined at 4 Å in 1976 and revealed a disordered Fc region, whereas a crystal structure at 3.4 Å of the isolated Fc region showed the carbohydrate acting as a bridge between the widely separated C_H_2 domains, in contrast to the more closely spaced Ig domains in the Fab (later deposited at 2.9 Å as PDB ID: 1FC1) (10). This structure was particularly important as it gave a structural view of a glycosylated protein that was to prove invaluable a few years later when visualizing and trying to interpret carbohydrates on viral glycoprotein antigens. The mouse Fab McPC603 structure at 3 Å with a small molecule ligand, phosphocholine (PDB ID: 2MCP), was for many years the prototypic example for understanding antibody–antigen recognition, where shape and electrostatic complementarity played key roles in the interaction in the antibody-combining site (11) (Fig. 2). The antigen-binding end of the antibody molecule was shown to be located in the variable domain (V_H_, V_L_) with its six complementarity-determining region (CDR) loops forming the antibody-combining site for interaction with antigen (Fig. 1). Thus, our initial insights into antibody structure and antigen recognition were fundamentally shaped by these early Fab structures with small molecules (reviewed (12)).

What was not clear at that time was how antibodies would interact with larger molecules such as proteins. We had to wait until 1986 and 1987 to get our first glimpses of antibody interactions with lysozyme (Fig. 2), which also was the prototypic antigen for protein crystallization and structure methods development, by the same groups of Poljak and Davies with crystal structures at 2.8-Å (later deposited at 2.5 Å as PDB ID: IFDL) and 2.5-Å (PDB ID: 2HFL) resolutions (13, 14). Structures of antibody complexes with influenza neuraminidase were then determined by Peter Colman and colleagues and deposited later as PDB ID: 1NCA, 1NCB, 1NCC, and 1NCD (15). These structures addressed many of the unresolved issues in the field as to whether the binding sites (epitopes) on the protein antigen were linear (consecutive amino acid stretches) or conformational (composed of multiple segments of amino acids) as most epitopes were found to be. The six CDRs in the light and heavy chains were more involved in interactions with proteins than with small molecules and, consequently, more of the antibody amino acids were involved in contacts with proteins (around 15–20) (16), although not all contacts contributed equally to the binding affinity and can vary from antibody to antibody (17). Rather than binding in cavities or grooves as for small molecules, the interacting surfaces for proteins tended to be much larger and more undulating.

Another key point of controversy in the 1970s and 1980s was whether the antibody–antigen interactions could be described by a lock-and-key mechanism, where neither the antibody nor the antigen changed conformation, or by induced fit, where the antibody or antigen, or both, molded themselves to the other partner to achieve a better fit. The prevailing view at that time was lock and key over induced fit, although the antibody–neuraminidase structure suggested more of a handshake, where antigen and possibly antibody changed conformation on binding (15). In 1991 to 1992, definitive proof for induced fit as the mechanism for some antibodies came from antibody structures with single-stranded DNA (PDB IDs: 1NBV,1CBV) and a peptide (PDB IDs: 1HIN, 1HIL) (18, 19). Thus, it seemed that both lock-and-key and induced fit, or aspects of both, were used, which may in retrospect not be surprising, especially with a diversity system like antibodies. From the accumulation of these pioneering studies on antibodies, many of the burning questions in the field had apparently been addressed on how antibodies, from a genetic and structural viewpoint, are able to recognize the enormous universe of potential antigens. Thus, what seemed to remain unresolved at the time were the fine details on how antibodies recognize specific antigens (20).

By the beginning of the 1990s, the difficulty in solving antibody structures had been largely overcome. It may not be that obvious now, but antibody Fab structures were quite challenging to solve in the 1970s and 1980s. When one of the authors (Wilson) entered the antibody field as an Assistant Professor in 1982, he talked to the giants in the field, Roberto Poljak and David Davies, to get a reality check on what was the likelihood of determining a new antibody Fab structure. It was somewhat dismaying to find that only 1 in around 25 antibody Fabs (Poljak) resulted in diffraction-quality crystals that led to subsequent structure determination, although another view (Davies) gave slightly better, but still daunting, odds of 1 in 5 to 1 in 10. Thus, it seemed that focusing on a single antibody or small set of antibodies was probably not the best approach. Thus, although we had initiated work on antibodies to influenza hemagglutinin and myohemerythrin peptides, our laboratory broadened our antibody projects to include HIV-1 gp120 V3 peptides, steroids, and diverse proteins. Crystallization was also a bottleneck even after one had painstakingly obtained Fabs from enzymatic cleavage of IgGs elicited in mice using hybridoma technology against the antigen of choice. Even if crystals were obtained, solving the structures required finding heavy atom derivatives for multiple isomorphous replacement using in-house X-ray sources at room temperature. It turned out that, by refining the antibody IgG cleavage and purification conditions, crystallizing the Fabs fortunately did not turn out to be as much of a problem as anticipated. Advances in antibody crystallization and methods, such as streak seeding and cross seeding (21, 22), helped coax antibody Fabs into forming well-ordered crystals. Thus, new methods for Fab structure determination were needed to now keep up with all of the Fab crystals, other than the usual trial and error methods with multiple isomorphous replacement. Molecular replacement (MR) as pioneered by Michael Rossmann (23, 24) was taking off, but antibody Fabs were quite flexible and MR proved challenging. Mirek Cygler and Wayne Anderson were the first to demonstrate that flexible Fabs could be solved by MR (25), and this finding further fueled antibody structure determination, along with new software for structure refinement (26), 2-dimensional area detectors (27, 28), gradually increasing access to synchrotron radiation facilities, and breakthroughs in cryocrystallography (29), which originated from a method that was devised to cool crystals of the ribosome to cryogenic temperatures for X-ray data collection by Håkon Hope and Ada Yonath (29, 30).

Since antibody structures were now being determined more frequently, it seemed that most pressing problems had been apparently solved, and David Davies, for example, largely exited from the antibody field to pursue other interests. However, a structure of an intact immunoglobulin with a normal hinge region was elusive as these flexible molecules were hard to crystallize. Structures of intact mouse IgGs appeared in 1995 to 1998 from Alex McPherson’s laboratory (PDB ID: 1IGT, 1IGY) (31, 32, 33). The first human IgG in 2003 (PDB ID: 1HZH) (34) further highlighted the asymmetry in the Y-shaped antibody molecule due to the flexible hinge region connecting the Fab domains to the Fc (Fig. 1). But it was not until human antibodies could be isolated or recombinantly expressed did we realize that structural and functional insights in the antibody field were far from over. Description of many new features, such as extra-long CDR H3 loops, large and small insertions and deletions in the antibody, posttranslational modifications such as tyrosine sulfation and glycosylation, would come from studies of how human antibodies responded to human pathogens, which we will return to in later sections.

We were also to find out that antibodies could come in other flavors. Single-Ig domain antibodies, also known as nanobodies, or V_H_H domains when derived from a heavy chain, are much smaller and can fit into smaller nooks and crevices on antigens (35). The first of these nanobody antibody structures was derived from a heavy-chain-only antibody discovered in camels (36, 37) and then in other members of the camelid family, such as llamas (36). The first llama nanobody structure at 1.85 Å in 1996 (PDB ID: 1HCV) showed that it adopted an Ig fold similar to that of a V_H_ in a conventional antibody, but with greater hydrophilicity and with only three CDRs available for antigen binding (38) (Fig. 1). These camelid V_H_H domains were also unusual in that they often contained extra disulfide bonds that could constrain their long CDR H3 loops to CDR H1 (39). Another nanobody structure in 1996 in complex with an antigen (PDB ID: 1MEL) showed how its long CDR3 (without an extra disulfide) could penetrate deeply into the active site of lysozyme confirming that these nanobodies could indeed access recessed sites (40) (Fig. 2). The use of only three binding loops compared with six in a conventional antibody did not seem to adversely affect specificity and potency. Cartilaginous fish such as nurse sharks also have heavy-chain-only antibodies termed immunoglobulin new antigen receptors that can also bind with high affinity and specificity to antigens, such as lysozyme (PDB IDs: 1SQ2, 1T6V) (41) (Fig. 1). The immunoglobulin new antigen receptor V_H_ domains have a very short CDR 2 and therefore use only two CDRs to attain high-affinity binding. Nanobodies from camelids and sharks as well as engineered human V_H_ domains are now being used extensively as reagents for research, immunodiagnostics (42), molecular imaging (43, 44), promotion of crystallization of a protein of interest (45, 46), as well as for their therapeutic potential (47), including against SARS-CoV-2 (48, 49, 50) as well as related coronaviruses (51). Nanobodies are selectable by vaccination or library panning and are generally very soluble and stable and can be produced in large quantities. Unusual features are also found in antibodies from other animals such as cows, where a subset of cow antibodies have a very long CDR H3 (60 residues or more) encoded primarily by a superlong D region that contains several disulfides (52) and forms an independent knob-shaped domain displayed on top of a long β ribbon stalk (PDB ID: 4K3E, 4K3D) (53) (Fig. 1). Why these particular antibodies are present in cows is not yet known. Antibodies from chickens (54) (PDB ID: 4GLR) also have additional Fab disulfide bonds in their CDR regions that are not often seen in human or mouse Fabs (55).

## Non-Ig fold antibodies

Jawless fish (agnatha) originated over 300 million years ago, and hagfish and lampreys are now the remaining examples. Conventional immunoglobulin domain-based antibodies as described above are not found in agnathans, but instead variable lymphocyte receptors (VLRs) are the key players in the adaptive immune response (56, 57, 58). VLRs are composed of multiple Leu-rich repeats, which, like the V(D)J elements of conventional antibodies, are highly variable in sequence and can be mixed and matched so as to adapt to a variety of antigens (Fig. 1). Carbohydrate, such as H-trisaccharide, was the first antigen to be visualized (PDB ID: 3E6J) in a VLR (Fig. 2). The carbohydrate binds in the hypervariable concave face of the VLR where one of the Leu-rich repeats is longer and extends over the antigen akin to CDR H3 in conventional antibodies (59). Protein antigens have also been observed to bind to VLR such as lysozyme (PDB 3G3B) (60) (Fig. 2), immunodominant glycoprotein of *Bacillus anthracis* spores (PDB ID: 3TWI) (61), TLR5 (PDB IDs: 6BXA, 6BXC) (62), influenza virus hemagglutinin (63), and other proteins and carbohydrates (64).

This same type of Leu-rich repeat fold has been co-opted into the mammalian innate immune system as Toll-like receptors (TLRs 1–11 in humans) with a single membrane-spanning region for recognition of specialized antigens from microbial pathogens. The first TLR structure in 2005 showed a horseshoe-like solenoid structure for the ectodomain of TLR3 that was assembled from 23 Leu-rich repeats (PDB IDs: 1ZIW, 2A0Z) (65, 66) (Fig. 1). A crystal structure of TLR3 with its double-stranded DNA ligand showed that 40 to 50 bases are required to span the TLR3 homodimer for signal transduction to trigger an anti-inflammatory response (PDB ID: 3CIY) (67) (Fig. 2). TLRs other than TLR3 turned out to be more difficult to express and purify, and innovative engineering by Jie-Oh Lee and colleagues to produce hybrid constructs of the TLR with some hagfish VLR repeats led to breakthroughs in structure determination of further TLRs, includingTLR4 (PDB ID: 2Z63) (68), TLR1-TLR2 with lipopeptide (PDB ID: 2Z80) (69), TLR5 with flagellin (PDB ID: 3V47) (70) (Fig. 2), TLR9 with DNA (PDB IDs: 3WPC, 3WPD, 3WPE) (71), and TLR8 (PDB IDs: 3W3K, 3W3L, 3W3M) and TLR7 (PDB IDs: 6LW1, 6LVY, 6LVZ, 6LW0) with single-stranded RNA and small molecule agonists (72, 73, 74). Thus, the Leu-rich repeat fold found in VLRs for general recognition of antigens in jawless fish evolved into specialized receptors that recognize components of microbial pathogens in the mammalian innate immune system. These TLRs act as an immediate defense system against microbial pathogens and, upon antigen recognition, activate signal transduction pathways to regulate inflammatory and other responses.

## Antigen receptors in cellular immunity

Antibodies in the adaptive humoral immune system are only one type of immune receptor that can recognize foreign antigens from pathogens or mutated antigens from cancer cells. The cellular side of the adaptive immune system has a more complex antigen recognition system that begins with the major histocompatibility complex (MHC), which is encoded by a diverse set of polymorphic genes. The MHC molecules first bind and then present antigens to T cell receptors, which are the cellular equivalent of antibodies, in a process known as MHC restriction (for a review see (75)). For many years, it was assumed that MHC molecules recognized intact antigens like antibodies. However, the diversity of MHC molecules is not as great as that of antibodies and it was not clear how they could recognize the universe of potential foreign antigens. This conundrum was solved in the 1980s when it was found that MHC molecules recognized processed antigens in the form of peptides (76, 77). Each MHC molecule is able to present a range of different peptides that contain certain conserved features that are specific to each MHC molecule.

The first structure of an MHC molecule, that of the human class I histocompatibility antigen HLA-A2, was determined in 1987 by Pamela Bjorkman and Don Wiley and colleagues and showed that the antigen-binding groove was formed by two long α helices that were supported on a β sheet platform (PDB ID: 1HLA) (78) (Fig. 1). The HLA molecule is formed from two chains: the α chain is composed of three domains, two of which form the MHC–antigen groove (MHC fold) atop an Ig-fold domain that associates with β_2_ microglobulin, also with an Ig-like fold. Within the MHC-binding groove, density for an antigen was found that was presumed to be a peptide or mixture of peptides (78, 79). Structures of MHC class I molecules with single peptides in 1992 revealed that the peptides bound in an extended conformation within a groove closed at both ends (Fig. 2). The MHC engaged in many interactions with the peptide backbone that would account for its specificity for peptide antigens (PDB ID: 2VAA, 2VAB, 1HSA) (80, 81, 82, 83, 84). A few conserved pockets in the groove provided specificity for particular amino acids (anchor residues) and enabled families of peptides with similar sequences containing appropriately placed anchor residues to be accommodated in these pockets. Thus, it was now clear how a limited number of MHC molecules could bind diverse peptides and how peptide length (~9 residues) was restricted by the finite length of the binding groove. How peptides bind to MHC class II molecules was answered with the structure of HLA-DR1 in 1993 with a mixture of endogenous peptides (85) and in 1994 with a single influenza virus peptide (PDB ID: 1DLH) (Fig. 1) and bacterial superantigen (PDB ID: 1SEB) (86) (Fig. 2). The MHC molecule in this case was formed as an α/β heterodimer with an open-ended groove where longer peptides could bind and spill over. Thus, the presentation of peptide antigens, derived from processing of viral antigens *via* proteasomes in the cytoplasm or from enzymes in endosomal/lysosomal compartments by the two major classes of MHC molecules could now be understood on a molecular level.

The next major question was how the T cell receptor (TCR) recognized this MHC–peptide complex (87). The αβTCR (PDB ID: 1TCR) turned out to look very much like the Fab of an antibody, where its variable Vα and Vβ domains adopted an Ig-fold with hypervariable regions corresponding to the antibody CDRs (87) (Fig. 1). The Cα domain deviated substantially from that in a conventional Ig but still paired with the Cβ domain. The TCR interacted with the MHC–peptide complex by binding diagonally across the top surface of the MHC-binding groove with respect to its long axis (PDB IDs: 1AO7, 2CKB) (87, 88, 89). The CDR3s from the α and β chains straddled the center of peptide and the other CDRs contacted MHC residues that accounted for specificity of the TCR for both peptide and MHC and thereby explained the structural basis of MHC restriction.

Other nonclassical MHC-like molecules such as CD1 also adopt an MHC-like I fold (PDB ID: 1CD1) (90) but with a much larger and more hydrophobic groove that can present other types of antigens, including glycolipids, such as α-galactosyl ceramide for CD1d, to invariant natural killer T cells (NKT cells) (Fig. 2). CD1b has the largest binding pocket that accommodates lipid and glycolipid antigens with long alkyl chains (PDB IDs: 1UQS, 1GZQ, 1GZP) (91, 92). A structure of CD1d complexed with a potent NKT cell agonist, α-galactosylceramide, revealed that the semi-invariant T cell receptor (NKT TCR) interacts in a more parallel than diagonal manner, and at one end of the CD1 antigen-binding groove, that then differs from αβ TCR recognition of classical MHC molecules with bound peptides (PDB ID: 2PO6) (93). An excellent review of TCR recognition of classical and nonclassical MHC molecules can be found in (94). A further specialized MHC molecule is HLE-E (MHC class 1b) that binds to conserved leader sequences from class Ia MHC molecules (PDB ID: 1MHE) (95) and acts as a checkpoint for NK cells. This brief summary here does not do justice to the other components of T cell recognition involved in T cell signaling that include CD4 and CD8 that define CD4 cells (T helper, *i.e.*, aids in the antibody response) and CD8 cells (T killer, *i.e.*, can directly kill infected cells) cells, which, respectively, bind MHC class I and II molecules. CD3 is also a major component of the T cell signaling complex, where the TCR interacts noncovalently with CD3γε, CD3δε, and CD3ζζ. A recent cryo-EM structure finally revealed a complete structure of the human TCR–CD3 complex in its octameric assembly of αβTCR:CD3γε:CD3δε:CD3ζζ with 1:1:1:1 stoichiometry (PDB ID: 6JXR) (96) (Fig. 2). For a comprehensive recent review of the structural basis of T cell activation, please see (97).

## Cytokine receptors

**Figure 3:**
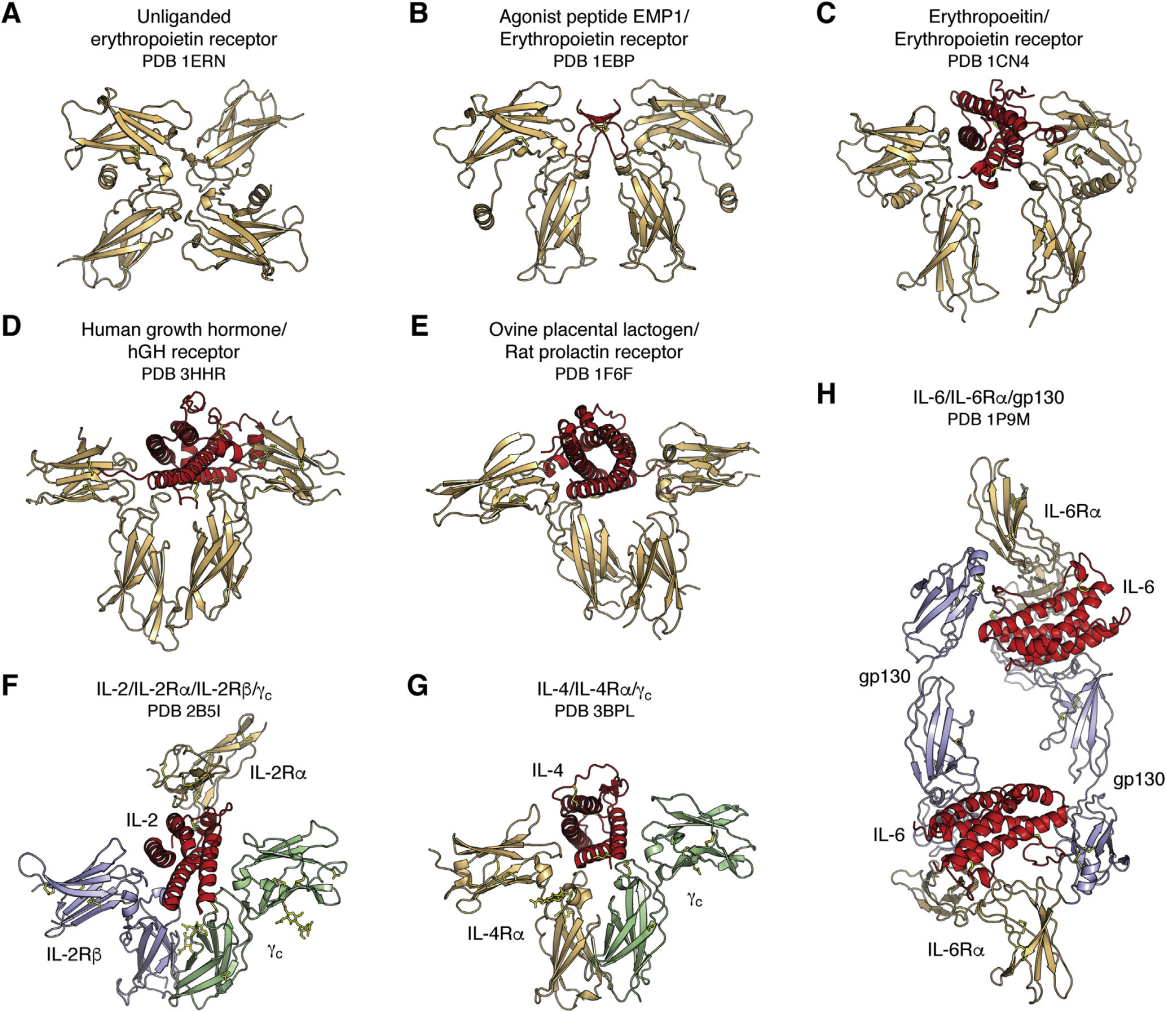
**Hematopoietic and type I cytokine receptors.** These signaling receptors have a self-ligand and can form homodimers (EPOR, *A*–*C*; hGHR, *D*; rat prolactin receptor, *E*), heterodimers (IL-4R, *G*), heterotrimers (IL-2R, *F*), and other high-order complexes (IL-6R, *H*) with their ligands. EPO receptors also exist as inactive preformed dimers (*A*) on the cell surface and change conformation to an active, signaling-competent state on interaction with their natural ligand (*C*). Preformed dimers have now been found in other cytokine receptors. Cytokine receptor α, β or gp130, and γ chains are colored *beige*, *blue*, and *green*, respectively, with the bound hormone or agonist colored in *red*.

A further set of receptors in the immune system are involved in cell differentiation, proliferation, and signaling. Different types of cytokine receptors can be found, and this topic is much too extensive to cover in detail here. So, a brief summary of class I cytokine receptors follows here where structural similarities, such as fibronectin type III superfamily domains and immunoglobulin-like folds, as well as other sequence motifs, are commonly found in their ectodomains. Family members can bind hormones (*e.g.*, growth hormone), prolactin, erythropoietin (EPO), colony stimulating factor or cytokines (interleukins), etc. and signal through interaction of their intracellular domain with the Janus kinase (JAK) family of tyrosine kinases. From an historical point of view, the crystal structure of human growth hormone (hGH) in complex with the ectodomain of the hGH receptor was an enormous breakthrough in the field and provided key insights into how this family of receptors is assembled and functions (PDB ID: 3HHR) (98). The hGH adopts a four-helix bundle with an unusual up–up–down–down topology (instead of the more usual up–down–up–down topology for other four-helical bundle proteins) and binds to two receptor chains that are each composed of two fibronectin III domains, but which are assembled in a very different way from antibodies into an L-shape (Fig. 3). The homodimeric hGH receptor forms a T-shape and binds to different faces of the hormone but nevertheless uses similar residues in each chain to bind to different residues on hGH but with different affinities (Fig. 3). This promiscuity in receptor recognition was fascinating and was reminiscent of another surprise in the 1990s where essentially the same epitope surface on influenza virus neuraminidase could bind to different antibodies (NC10, NC41) in different binding modes and with different contact residues and chemical interactions (PDB IDs: 1NMB, 1NCA) (99). Further promiscuity is seen in the prolactin receptor that can bind not only prolactin but can be activated also by hGH and human placental antigen. A structure of ovine placental lactogen with the ectodomain of the rat prolactin receptor showed binding to either side of ovine placental lactogen but with more asymmetry in receptor residues used in each interface that may facilitate binding to different ligands (PDB ID: 1F6F) (100) (Fig. 3).

It was also proposed that ligand-induced dimerization of cytokine receptors brought the two subunits together for signal transduction *via* the membrane and intracellular components of the receptor in the JAK-STAT pathway. Thus, there was great interest in the receptor-binding residues that interact with the ligand and would enable receptor dimerization. Although around 30 residues in hGHR are involved in binding in each chain to each side of the hormone, only a subset of these residues are important for the binding affinity. These so-called hot-spot residues defined a smaller functional epitope that is dominated by a central hydrophobic region surrounded on the periphery by more polar residues that can provide specificity (101). The structure of the erythropoietin receptor (EPOR) showed a similar overall fold but, in this case, a novel aspect was that it was bound to a small molecule agonist, a 20-residue cyclic peptide EMP1 that had been identified from a phage display library (PDB ID: 1EBP) (102). The peptide and receptor formed a symmetric dimer that differed from the more asymmetric hGHR complex (Fig. 3). It was not expected at that time that a small molecule agonist would be able to mimic a protein hormone, but peptide dimerization enabled formation of a functional dimeric receptor signaling complex (102). The largely hydrophobic peptide epitope corresponded to the smaller functional epitope on hGHR and, hence, EMP1 represented what was regarded as a “minimized” hormone. When the EPOR structure with its natural EPO ligand was determined, the complex was much more asymmetric with a 120-degree relationship of its two receptor chains to maximize signaling through the intracellular kinase pathways (PDB IDs: 1EER, 1CN4) (103) (Fig. 3). A final surprise for the EPOR was that it existed as a preformed dimer on the cell surface and hence contradicted the ligand-induced dimerization model (PDB ID: 1ERN) (104, 105) (Fig. 3). Ligand binding then altered the relative orientation of the two chains of the receptor and decreased the distance between the intracellular signaling domains for interaction with JAK2 and, hence, went from an inactive to an active signaling state. In another twist, the same hot spot residues that were used to interact with the ligand hormone are used for the receptor homodimer interactions, thus again illustrating the plasticity of the receptor in binding to different ligands, whether the natural EPO ligand, small molecule agonist, or the receptor itself.

Receptors in the interleukin family of cytokine receptors can be more complex and be composed of different types and numbers of chains for assembly of their signaling complexes. The IL-2 receptor is a prototypic example with multiple chains. The IL-2 receptor was initially thought to consist of one α chain, the so-called Tac antigen, but was later found to bind not only a β-chain but also a common γ chain (γ_c_) that is found in other IL receptors (106, 107) (for an excellent review of cytokine receptors with shared β, γ and gp30 chains, please see (107)). The structure of this heterotrimeric receptor remained elusive for many years and was determined in 2005 (PDB ID: 2B5I) (108) (Fig. 3). Beautiful crystals of the p55 Tac antigen with IL-2 were obtained in our laboratory in 1989 (109), but it took us until 2006 to determine the IL-2 plus ternary IL-2R complex structure (PDB ID: 2ERJ) with MR help from the 2B5I structure (110). Thus, the IL-2 receptor proved to be a moving structural target with new chains being discovered that then required expression and assembly of these different chains with IL-2. Technology often has to change to make progress, and the moral of the story here is that it can still be productive and rewarding to not give up on a structure even if it takes almost 20 years!! The different chains in the heterotrimer can assemble into low- and high-affinity complexes. The high-affinity complex is formed between the β and common γ chain, and IL2-Rα’s role thus seemed to be to deliver IL-2 to the β and γ chains for assembly of a 1:1:1:1 complex. Thus, this general family of receptors can be quite diverse and can form homodimers, heterodimers, heterotrimers, and higher-order assemblies. Identical chains (γ_c_, gp130) can be shared with other receptors in the cytokine family (see Fig. 3). This review cannot begin do justice to all of these receptors, but these examples give some idea of what has been learned over the years. The crystal structures revealed use of a common fibronectin type III fold in each of the receptors that formed the heart of the interaction with their respective ligands. Other excellent reviews provide further and more comprehensive information on the different classes and types of cytokine and hematopoietic receptors (107, 111).

## Antigen structure

**Figure 4:**
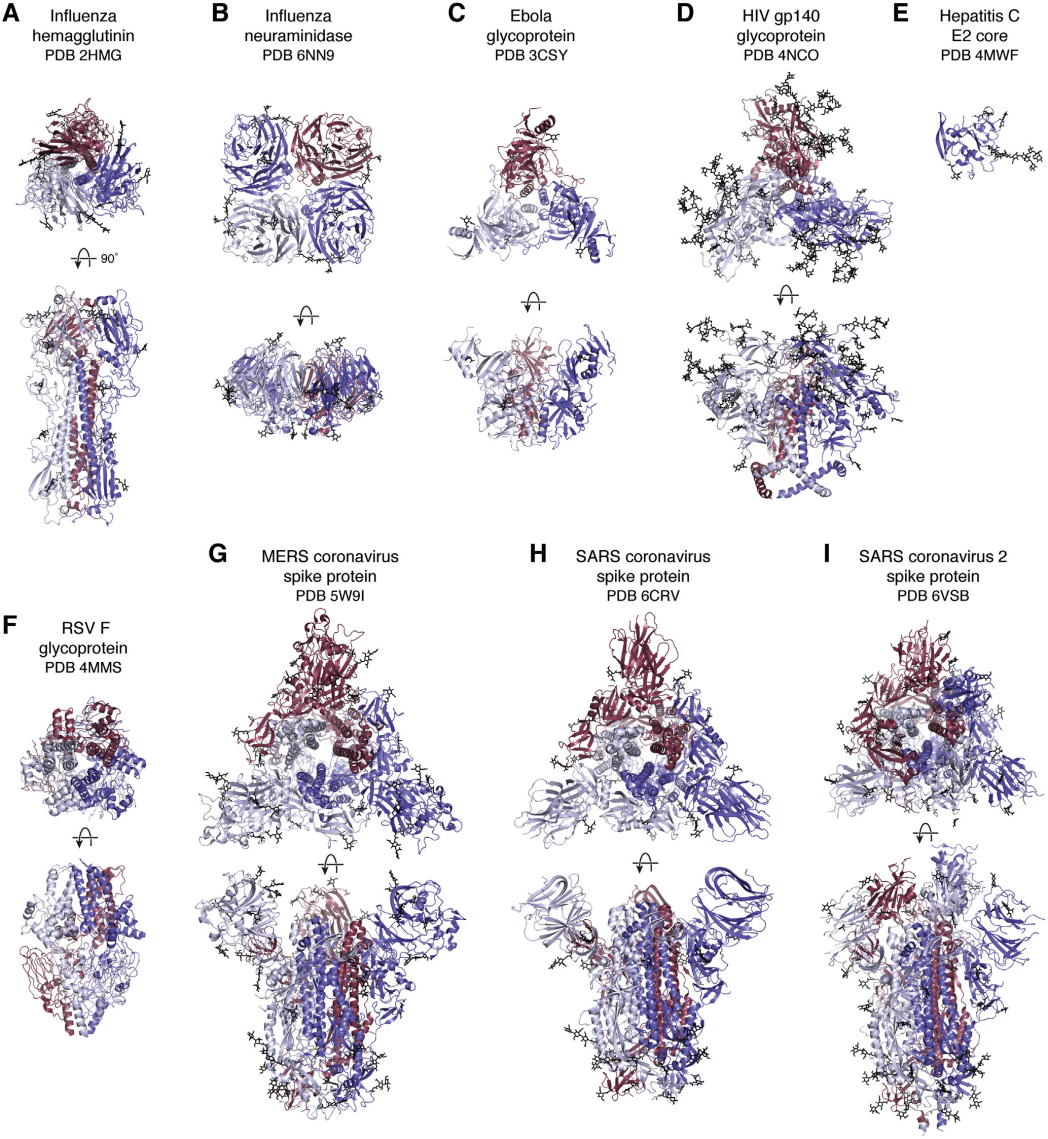
**Viral antigens on enveloped viruses.** Viral glycoproteins embedded in the viral membrane are responsible for entry of viruses into host cells. These envelope proteins contain receptor-binding sites and membrane fusion activities or are involved in progeny release and cell egress (influenza neuraminidase). Many of these viral antigens are homotrimers (*e.g.*, influenza HA (*A*), Ebola virus GP (*C*), HIV-1 (*D*), RSV F (*F*), MERS (*G*), and SARS-CoV 1 and 2 spike proteins (*H* and *I*)), whereas Influenza NA (*B*) is a homotetramer. The HepC E1E2 glycoprotein is thought to exist as a heterodimer, but only the structure for the monomeric E2 core has been determined (*E*). These antigens are usually heavily glycosylated to shield themselves from antibodies in the immune system. In all panels, the individual subunits of each glycoprotein are colored *red*, *white*, or *blue* and *top* (looking down the trimer or tetramer axis) and side views where the viral cell membrane would be on the *bottom* are shown for each. Carbohydrate is shown in *black*.

The first structure of a viral antigen from an enveloped virus was reported in 1981 (112). The influenza virus hemagglutinin (HA) is the major surface antigen on the virus and enables cell entry through its receptor binding and fusion activities. The HA is a heavily glycosylated protein with ~25% carbohydrate by weight. The humoral immune response is also directed toward the HA on influenza virus. The HA structure from the pandemic H3N2 1968 virus showed an interesting architecture with a globular head domain that encompasses the receptor-binding site and an elongated helical stem domain that houses the fusion machinery (PDB IDs: 1HMG refined later as 2HMG, 3HMG, 4HMG, 5HMG). Like many viral antigens on enveloped viruses, HA is a homotrimer (Fig. 4). Through analysis of natural variation that arose after introduction of the pandemic H3N2 Hong Kong virus into the human population in 1968 and from laboratory escape mutations, it was possible to visualize for the first time the major antigenic sites on a virus (113). Thus, this HA structure proved to be highly informative on how it functioned, how it could escape from the immune system and, hence, why seasonal flu vaccines are required on an annual basis.

The structure determination itself was of interest as it represented a very large protein at the time (~200 kDa) and not many glycoproteins had been worked on structurally with the Fc region of antibodies being an exception (PDB IDs: 1FC1, 1FC2) (10). The crystallographic experience with glycans on the Fc combined with NMR structures of glycans (114, 115), along with valuable conversations with Hans Deisenhofer and Jeremy Carver, proved extremely valuable for helping interpret glycans on the HA. Building structures in the computer rather than by stacking density maps in a Richards’ box had really only just started thanks to the development of FRODO by Alwyn Jones (116, 117) and BILDER by Bob Diamond (118) and modified by Bob Ladner, MRC Cambridge. It was challenging enough at the time to build polypeptides using the computer without having to think about how to include carbohydrates. Computation itself was another problem, and the Evans and Sutherland graphics system attached to VAX computers that became available in the late 1970s enabled interactive building into electron density maps to take off, although the model building process still took several months. One of the authors spent several months to complete the model of trimeric HA glycoprotein with help from Bob Ladner in customizing BILDER. However, at the end, one had a structure with atomic coordinates without the need to wonder how long it would take to generate such coordinates from the wire models previously built in Richards’ boxes. Refinement of the HA structure had to wait until 1990 (26, 119) as there were far too many atoms to handle with available computers and software in 1981. The crystal structure of the other main antigen on influenza virus, the neuraminidase (NA), followed soon thereafter in 1983 from Peter Colman and colleagues (120, 121) for N2 NAs from 1957 and 1967 viruses. Influenza NA is a tetramer, and its enzymatic activity enables progeny virus to be released from infected cells by cleaving off sialic acid and thereby destroying the receptor for the HA. Progeny viruses can then escape to infect new cells. Thus, 1981 to 1983 represented a significant breakthrough in understanding the structure of viral glycoproteins, how they function, and how they escape from the immune system.

It is important to reflect on one of the main reasons that these two viral glycoproteins could be structurally characterized at that time. The glycoproteins could be cleaved off the virus surface by enzymes, such as bromelain (HA) and pronase (NA), from large amounts of virus grown in chicken eggs. For many years, influenza HA and NA structures were determined in this way from protein extracted from virus. For later work on HAs from highly pathogenic viruses, such as the pandemic 1918 H1N1, it became necessary to recombinantly express and purify the glycoproteins. A baculovirus-insect cell expression system was developed in the early 2000s by James Stevens in our laboratory to produce sufficient quantities of 1918 H1 HA (PDB ID: 1RD8) (122) and H5N1 HA (PDB ID: 2FK0) (123) for structure determination; the expression system was subsequently refined by Damian Ekiert (124) and has largely remained the workhorse in many laboratories throughout the world for HA production. It might seem surprising today that such a breakthrough in a recombinant protein production system was so beneficial, but it was not easy to produce large quantities of HA, unless one had access to vast numbers of chicken eggs and influenza expertise in a virology laboratory. Sir John Skehel at The National Institute for Medical Research, Mill Hill, London, had pioneered cleavage and extraction of antigens from influenza viruses (125, 126) that resulted in the first HA structure.

The next advance in 1988 was the structure of HA in complex with its receptor; the sialic acid receptor could then be visualized binding in a shallow receptor-binding pocket at the apex of the HA trimer (PDB IDs: 5HMG, 4HMG) (127). Much of the history of influenza HA structural studies can be found in (128) and a recent update this year in (129).

**Figure 5:**
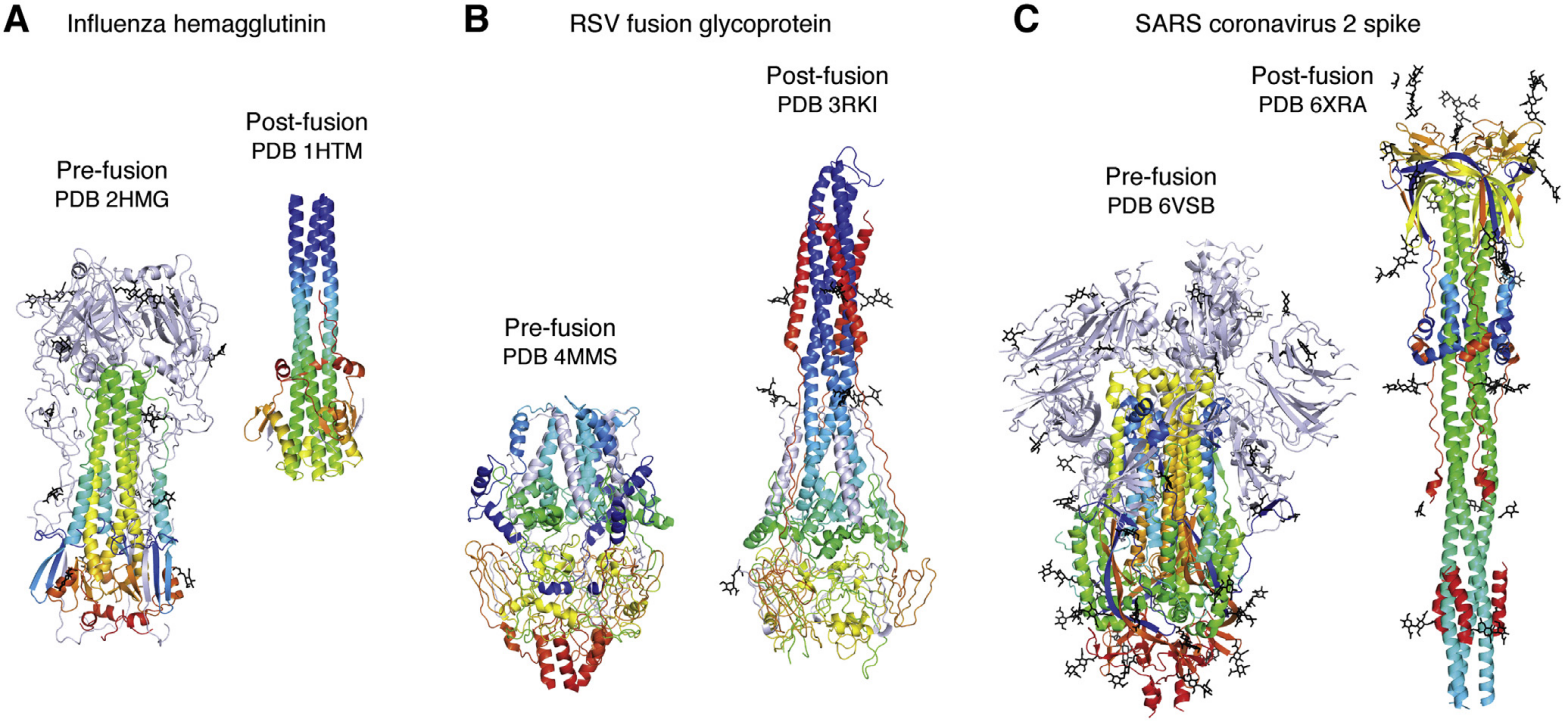
**Spring-loaded conformational rearrangements in viral glycoproteins to attain their fusogenic state.** Viral antigens involved in cell entry are metastable and undergo large conformational changes to attain their fusion-active form after engaging their receptor(s). Prefusion (*left*) and postfusion (*right*) structures of (*A*) Influenza hemagglutinin, (*B*) RSV fusion glycoprotein, and (*C*) SARS-CoV-2 spike are illustrated with the fusion portions of the proteins colored in a rainbow with *blue* and *red* corresponding to the N and C termini. Molecules are oriented so that the viral membrane would be on the *bottom* and target cell membrane on the *top*.

Other viral glycoproteins proved very difficult to produce compared with the initial successes with influenza virus. A particularly challenging viral antigen was that of the HIV-1 Envelope protein (Env). Many groups worldwide tried for years to express and purify trimeric Env for structural and functional studies, but it proved a gargantuan task. Viral glycoproteins from enveloped viruses are inherently metastable so as to perform one of their essential biological functions to fuse the viral membrane with the host cell membrane. This fusion activity enables the viral genetic material to enter the host cell, and fusion must be initiated at the right place and at the right time. Influenza HA, like other viral fusion proteins is synthesized as a single precursor polypeptide chain (HA0) that is cleaved into two chains (HA1, HA2), thus exposing the fusion peptide at the N terminus of the second chain. In its prefusion form, the HA keeps both chains together by an interchain disulfide. Only after binding to target cells in the respiratory tract, followed by viral entry into the cell *via* endocytosis, does the low pH in the endosome trigger major conformational rearrangements to enable the HA to adopt its fusion active and postfusion forms (Fig. 5).

For HIV Env, gp160 is cleaved into gp120 and gp41 with no interchain disulfide to help stabilize the prefusion form. Thus, producing Env protein turned out to be extraordinarily challenging. John Moore and colleagues from the early 1990s relentlessly pursued Env production for structural studies and, by testing many different mutations, discovered two that stabilized the trimer of gp120/gp41: a disulfide that kept both chains together (130) and an isoleucine to proline mutation in gp41 thought to help strengthen gp41–gp41 interactions and reduce flexibility required for conversion to the postfusion form (131). A further alteration at the gp120–gp41 boundary in gp160 aided cleavage by furin and further enabled these combined mutations to stabilize the Env trimer in a native-like conformation. Further refinements were achieved by screening many different Env constructs and analyzing their ability to adopt well-formed trimer configurations using negative-stain electron microscopy in Andrew Ward’s fledgling laboratory (132). The end result was selection of a viral Env sequence that ultimately proved amenable for structural studies (133, 134), and this journey has been documented in recent review papers (135, 136). This breakthrough culminated in atomic-level cryo-EM and X-ray structures of the Env protein in 2013 (PDB IDs: 3J5M, 4NCO) (137, 138). It should be noted that this cryo-EM structure slightly preceded the current EM resolution revolution and did not use a Titan Krios. Higher-resolution crystal structures of Env then followed (PDB IDs: 4TVP, 5CEZ) (139, 140). For the X-ray and EM studies, complexing the Env trimer with Fabs derived from neutralizing antibodies proved beneficial. For the X-ray studies, the Fabs facilitated lattice formation and helped cope with the glycan heterogeneity as well as conformational flexibility in the variable loops of Env. For EM, the Fabs provided additional mass and features to help align the single particles used in the reconstructions. An added advantage of the EM studies was that the partial deglycosylation used to reduce glycan heterogeneity as well as expression in cell lines that promote more uniform glycoforms (*i.e.*, high-mannose glycans) for crystallography was not required. Hence, one could determine EM structures with fully native glycans where Env was expressed in cell lines that produce both high-mannose and complex sugars more akin to those on the virus. Another advantage of modern-day EM is that much less starting material is required compared with crystallography.

In order to advance structural work on other viruses, stabilization of their Env glycoproteins was also absolutely essential. The respiratory syncytial virus (RSV) fusion (F) glycoprotein transitions readily to its postfusion form (141), but stabilization by antibodies that specifically recognize the prefusion form enabled the prefusion structure to be determined (PDB ID: 4JHW) (142) (see also Fig. 5).This crystal structure then enabled the design of mutants to stabilize the prefusion form, which also increased its expression level, and led to an RSV vaccine candidate that could protect animals against viral challenge (PDB IDs: 5C6B, 5C69) (143). The RSV work was a fantastic demonstration of proof of concept for structure-based vaccine design (144) and has become the poster child for rational vaccine design.

Another important and deadly human pathogen is the Ebola virus. The structure of its glycoprotein (GP) in its prefusion form bound to a neutralizing antibody was determined in 2008 (PDB ID: 3CSY) (145). The Ebola virus GP is also a trimer but has unusual features such as a glycan cap and a large mucin domain that sits atop the GP and restricts access to the receptor-binding site as well as to antibodies. Cleavage of the mucin-like region and glycan cap during viral entry exposes more of the GP so it can function as other viral glycoproteins in the infection process.

Although several other examples of viral glycoproteins could be mentioned, the initial work on coronaviruses (CoV) has taken on special significance since the pandemic SARS-CoV-2 outbreak. Pioneering structural work on the human spike proteins from CoVs by single particle cryo-EM started with the spike structure from the seasonal HKU1 CoV (5I08) (145) and was then followed by MERS-CoV (PDB IDs: 5X5C, 5X5F, 5X59) (146, 147) and SARS-CoV spikes (5X58, 5X5B) (146). The trimeric spike is composed of the S1 and S2 domains. A very interesting feature of the S1 domain that differentiates it from other viral glycoproteins is that the receptor-binding domain (RBD) in the S1 domain can adopt up and down conformations that expose and mask the receptor-binding site, respectively. In MERS, most spikes seem to adopt RBD one up or two up conformations (147). Binding of neutralizing antibodies and receptor itself can also stabilize the other states. One very important advance at that time was redesign of the spikes to improve their stability and expression. A similar region in the spike protein was targeted for Pro substitutions as in HIV-1 Env. Emanating from the structural CoV work in the Ward and McLellan laboratories in collaboration with Barney Graham at the NIH VRC, two proline mutations were targeted to the loop between the first heptad repeat (HR1) and long central helix in S2 that prevented premature conversion to the postfusion form with the beneficial added effect of increasing the expression yield of prefusion spike trimers (147).

This 2P mutation was to prove invaluable for rapidly producing a structure of the spike protein for SARS-CoV-2, as well as for the different constructs used to produce the current vaccines and vaccine candidates. Indeed, in only a month after release of the genomic sequence of SARS-CoV-2, the first structure of its spike protein appeared using cryo-EM (PDB ID: 6VSB) (148), followed shortly thereafter by a second structure (PDB IDs: 6VYB, 6VXX) (149). A further stabilized version of SARS-CoV-2 spike protein was then designed and engineered by Jason McLellan and colleagues with six proline substitutions (HexaPro) that is currently the standard for producing prefusion-stabilized SARS-CoV-2 spikes (PDB ID: 6XKL) (150). Cryo-electron tomography (151, 152) enabled visualization of the spikes on the SARS-CoV-2 virion itself. Both pre- and some postfusion structures (Fig. 5) have been observed on the virus surface, as well as up and down conformations of the RBD. The spike density is relatively sparse compared with the ubiquitous cartoon versions of virus with its red spikes that appear frequently in the scientific literature and media. This SARS-CoV-2 spike density is greater than that of the Env protein on HIV-1 but much less than of the HAs on influenza virus. The spikes also seem to be quite flexible with three articulated hinges at the trimer base just before the ectodomain would enter the membrane. Thus, the spikes can adopt a wide range of orientations on the virus surface including bent over conformations rather than the conventional representation as straight-up with the trimer 3-fold axis perpendicular to the membrane surface (153). One of the protein-based vaccine candidates has also been visualized by cryo-EM (PDB IDs: 7JJI, 7JJJ) (154). The spike protein in the Novavax vaccine is formulated as a nanoparticle in polysorbate 80 detergent and has some slight differences in its S1 structure compared with the soluble ectodomain structures. Interesting features include interactions between spike trimers that enable formation of higher-order complexes that may be important for their function. Close proximity of at least some trimers can enable antibodies to span from one trimer to the next and enhance antibody affinity through avidity where both Fab arms of the IgG can interact simultaneously on the viral surface.

Thus, we now have amassed a vast amount of information about the structure and function of antigens from enveloped viruses. Almost all are homotrimers, although influenza NA is a homotetramer and HCV E1E2 is a heterodimer. These oligomers enable multivalent binding to their natural receptors and, hence, enhance avidity and protect to some extent against any decrease in fitness that might arise from incorporating escape mutations in and around the receptor-binding site. All of these viral antigens are heavily glycosylated to protect against the humoral immune response by limiting the amount of exposed polypeptide surface on the protein (*e.g.*, HIV-1 Env has 81 or more glycans per trimer) (for recent papers and reviews see (155, 156, 157)). Other viruses have fewer glycans that protect the protein surface to different extents (158, 159). We thus know much more now about the various roles and nature of glycans on the virus surface. Recent breakthroughs in the analysis of site-specific glycosylation (158, 160, 161) have increased our knowledge of their effects on antibody recognition and their involvement in vulnerable sites (major epitopes) on the virus (for recent papers and reviews see (155, 157, 159, 162)).

As alluded to above, another feature of these viral glycoproteins is their metastability. The viruses first of all have to bind to their target cells. After binding to a receptor and sometimes also to a coreceptor, incredible conformational changes are triggered in the viral glycoproteins to attain their fusion active and postfusion forms. The location of these events differs from virus to virus with fusion occurring either on the cell surface or in endosomal/lysosomal compartments, where the conformational rearrangements can be triggered by low pH, such as for influenza virus. The RBD of the Env protein can also be shed, such as in HIV Env and SARS-CoV-2 spike protein, to facilitate the fusion process. The large conformational rearrangements from prefusion to postfusion structures are illustrated in Figure 5 for three viral fusion proteins. The first postfusion structure observed was for influenza HA, where the stem domain extensively rearranges and elongates to form a six-helix bundle; this spring-loaded mechanism enables the fusion peptide to end up next to the host cell membrane for initiation of the fusion process (PDB ID: 1HTM) (163, 164). Similar postfusion structures have been visualized for the RSV F protein (PDB IDs: 3RKI, 3RRT) (141, 165), gp41 of HIV-1 (PDB ID: 1AIK) (166), Ebola virus GP2 (PDB ID: 1EBO) (167), and SARS-CoV-2 S2 protein (PDB ID: 6XRA) (168).

## Antibodies to SARS-CoV-2

Extensive work over many years to determine structures for antibodies in complex with viral antigens provided the methodology and tools that enabled rapid progress on antibodies to SARS-CoV-2. Significant breakthroughs in the isolation of human antibodies from single B cells occurred in the late 2000s (169, 170, 171) that enabled many more antibodies to be isolated for structural and functional studies as well as therapeutic applications. Many recent excellent reviews on isolation of antibodies from natural infection or vaccination (169, 171, 172, 173) can be consulted as well as reviews covering the analysis of antibody structure and vulnerable neutralizing epitopes on viral antigens (174, 175) and structure-based vaccine design and antibody therapeutics (176, 177, 178, 179, 180, 181).

Here we focus on the breathtaking speed of accumulation of structural information on SARS-CoV-2 and interaction with antibodies from the start of the pandemic. The SARS-CoV-2 coronavirus appears to have its origins in 2019, although its exact etiology is still being discussed and debated. A novel coronavirus was first reported to the World Health Organization as the cause of pneumonia of unknown etiology on December 31, 2019. On January 11, Chinese scientists disclosed the full genetic sequence of SARS-CoV-2 (see: https://virological.org/t/novel-2019-coronavirus-genome/319 and https://www.sciencemag.org/news/2020/01/chinese-researchers-reveal-draft-genome-virus-implicated-wuhan-pneumonia-outbreak). On January 30, the World Health Organization announced a public emergency of International concern and on March 11 officially declared a global pandemic. Researchers around the world reacted en masse as soon as the virus sequence was released. Indeed, it only took just over a month for the first cryo-EM structure of the viral spike protein to appear in *bioRxiv* (182) and then in *Science* (148), followed by another cryo-EM structure in *Cell* (149).

**Figure 6:**
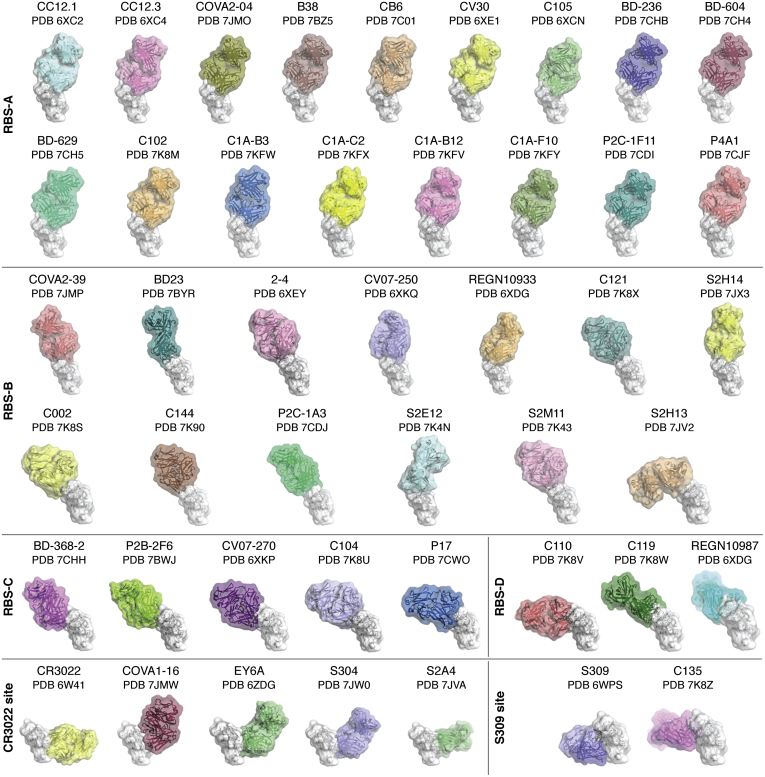
**Antibody recognition of the RBD of SARS-CoV-2.** A large number of neutralizing antibody structures to SARS-CoV-2 and related viruses have been determined in a relatively short time. Most of these antibodies (as depicted here) bind to the receptor-binding domain. These antibodies have been classified into regions that they bind on the RBD by Meng Yuan, Nicholas Wu, and Hejun Liu in the Wilson laboratory. Structures of the RBD (*white*) in complex with four groups of antibodies to the receptor binding site (RBS): RBS-A-targeting antibodies: CC12.1 (PDB ID: 6XC2) (186), CC12.3 (PDB ID: 6XC4) (186), COVA2-04 (PDB ID: 7JMO) (210), B38 (PDB ID: 7BZ5) (211), CB6 (PDB ID: 7C01) (212), CV30 (PDB ID: 6XE1) (213), C105 (PDB ID: 6XCM) (187), BD-236 (PDB ID: 7CHB) (214), BD-604 (PDB ID: 7CH4) (214), BD-629 (PDB ID: 7CH5) (214), C102 (PDB ID: 7K8M) (188), C1A-B3 (PDB ID: 7KFW) (215), C1A-C2 (PDB ID: 7KFX) (215), C1A-B12 (PDB ID: 7KFV) (215), C1A-F10 (PDB ID: 7KFY) (215), P2C-1F11 (PDB ID: 7CDI), P4A1 (PDB ID: 7CJF); RBS-B-targeting antibodies: COVA2-39 (PDB ID: 7JMP) (210), BD23 (PDB ID: 7BYR) (216), 2 to 4 (PDB ID: 6XEY) (190), CV07-250 (PDB ID: 6XKQ) (217), REGN10933 (PDB ID: 6XDG) (218), C121 (PDB ID: 7K8X) (188), S2H14 (PDB ID: 7JX3) (219), C002 (PDB ID: 7K8S) (188), C144 (PDB ID: 7K90) (188), P2C-1A3 (PDB ID: 7CDJ), S2E12 (PDB ID: 7K4N) (220), S2M11 (PDB ID: 7K43) (220), S2H13 (PDB ID: 7JV2) (219); RBS-C-targeting antibodies: BD-368-2 (PDB ID: 7CHH) (214), P2B-2F6 (PDB ID: 7BWJ) (221), CV07-270 (PDB ID: 6XKP) (217), C104 (PDB ID: 7K8U) (188), P17 (PDB ID: 7CWO) (222); RBS-D-targeting antibodies: C110 (PDB ID: 7K8V) (188), C119 (PDB ID: 7K8W) (188), and REGN10987 (PDB ID: 6XDG) (218). Cross-neutralizing antibodies are targeted to two sites, which are more highly conserved than the receptor-binding site: the very highly conserved CR3022-binding site: CR3022 (PDB ID: 6W41) (184), COVA1-16 (PDB ID: 7JMW) (223), EY6A (PDB ID: 6ZER) (224), S304 (PDB ID: 7JW0) (219), S2A4 (PDB ID: 7JVA) (219), and those targeting the moderately conserved S309 site that include an N-linked glycan: S309 (PDB ID: 6WPS) (189) and C135 (PDB ID: 7K8Z) (188).

At the outset, no antibodies were available for study from patients with SARS-CoV-2. Thus, the only option was to investigate antibodies to the previous SARS-CoV pandemic in early 2000. One of these antibodies, CR3022 (183), also bound to SARS-CoV-2, and its crystal structure showed that it targeted a highly conserved cryptic site in the RBD of the spike protein (PDB ID: 6W41). However, this binding site is only exposed when the RBD is in the up conformation (184). As the pandemic took off, it became possible to isolate antibodies from convalescent patients (see review and references therein (185)). Most of the neutralizing antibodies appeared to target the RBD with some against the N-terminal domain (NTD) (see refs. 5–20 in ref. (184)). What was immediately surprising was that these antibodies had sequences that were very close to germline with almost no somatic mutations but still had high affinity for antigen in the nanomolar range. The antibodies were also highly dominated by particular germline families (186, 187) that suggested that our humoral immune repertoire had off-the-shelf antibodies ready to spring into action. This paucity of somatic hypermutations was very encouraging for attempting to elicit such antibodies by vaccination. It is remarkable how many antibody structures to SARS-CoV-2 have already been deposited in the PDB and a snapshot of some of these antibodies against the RBD is illustrated in Figure 6. A more detailed review of the structure and properties of these antibodies can be found in (185, 188). It is clear now that three major binding sites for antibodies can be found on the RBD. Most antibodies target the receptor-binding site (RBS) at the top of the RBD but can do so in different ways. At least four modes of binding (A–D) can be observed to date (Fig. 6) where antibodies are able to approach the RBD with different approach angles. Antibodies to the SARS-CoV-2 RBS tend to be highly restricted in their binding and neutralizing properties to that virus and not to related viruses. This observation is explained as the ACE2 receptor-binding site on the RBD is not highly conserved between SARS-CoV and SARS-CoV-2, where only 7 of 16 binding residues are identical. The other two main sites for antibody binding are more conserved, especially the CR3022 site (24 of 28 residues), and are located on opposite sides of the RBD below the RBS (Fig. 6). Antibodies to these sites tend to be cross-reactive within viruses in the sarbecovirus family that includes SARS-CoV-2, SARS-CoV, and related bat and pangolin viruses. Hence design of vaccines or antibody cocktails that target these sites (CR3022, S309 (189)) may be valuable for consideration not only for the current SARS-CoV-2 pandemic but also to protect against future pandemics. Antibodies have also been found to the NTD and can also recognize the NTD when the RBD is in the down formation (*e.g.*, (190, 191)). These NTD antibodies are not cross-reactive with other SARS-like viruses. Extending vaccine or antibody cocktails to other betacoronaviruses, such as MERS, or to seasonal coronaviruses that include both alphacoronaviruses (NL63, 29E) and betacoronaviruses (HKU1, OC43) will likely require discovery of antibodies to the more highly conserved S2 (30%–40% sequence identity). One of the main concerns at present are the mutations that are arising in the spike protein and, in particular, in the RBD and NTD. Several of the mutations in the viruses described in the new United Kingdom, South Africa, and Brazilian lineages (192, 193, 194, 195, 196, 197) are in the heart of the antibody-binding sites. These mutations can affect natural immunity or vaccine-induced immunity and the antibody cocktails and plasma that are being used to treat patients with COVID-19. As more and more people are infected, and the use of vaccines and therapies become more widespread, further mutations may arise, but they are likely to be less frequent as vaccination levels rise. It is likely that updated vaccines akin to seasonal flu vaccines will have to be considered as the pandemic evolves.

## Conclusions

The last 50 years have been extraordinarily eventful in structural immunology. From modest but momentous beginnings starting with antibody structures in the 1970s, gradually more and more structural information emanated on the adaptive and innate responses to microbial pathogens as well as on receptors that regulate the immune and inflammatory responses. The impact of structural immunology is highlighted by 4530 antibody structures and 39,683 structures related to the immune system (of 173,537 total structures) with coordinates deposited in the PDB (as of January 13, 2021). It has therefore not been possible to cover all aspects of structural immunology and, hence, only certain topics have been highlighted here; our apologies if your favorite immune molecule was not included. During these 5 decades, many advances have been made in structural methods, crystallization, computation, synchrotron beamlines, cryocooling, and all of the associated equipment and tools that enable such development to take place. Structural genomics centers, such as the JCSG (198, 199, 200) that one of the authors was involved in, also contributed substantially to methods, tools, and equipment development to increase the throughput of structure determination, as well as deposition of large numbers of novel structures in the PDB through the Protein Structure Initiative of the NIH National Institute of General Medical Sciences (201, 202, 203, 204, 205, 206, 207, 208, 209). The PDB itself started modestly and has evolved over the years into an integral and indispensable resource that has taken on more and more key roles and functionalities that impinge on structure determination, structure validation, and outreach as well as acting as a repository to preserve structural data for eternity. The availability and utility of these structures have enabled structure-based vaccine design as well as engineering that has resulted in a wide range of antibody therapeutics and regulatory molecules. Indeed, many of the most effective, widely used, and highest-earning drugs are antibodies. It is therefore expected that the next 50 years will continue to prove exciting, eventful, and exceedingly productive for both structural immunology and the PDB.

## Conflict of interest

The authors declare that they have no conflicts of interest with the contents of this article.
